# P-1818. Measles Testing in a Large Commercial Laboratory in the United States, 2023-2025 in an Era of Increasing Measles Infections

**DOI:** 10.1093/ofid/ofaf695.1987

**Published:** 2026-01-11

**Authors:** Charles M Walworth, Laura Gillim, Suzanne Dale, Sharon K Martens, David Alfego, Ato Aikins

**Affiliations:** Monogram Biosciences/LabCorp, Laguna Beach, CA; Labcorp, Canadaigua, NY; Labcorp, Canadaigua, NY; Monogram Biosciences, San Francisco, California; Labcorp, Canadaigua, NY; Labcorp, Canadaigua, NY

## Abstract

**Background:**

Within the first 4 months of 2025, more than 800 confirmed cases of measles were reported by 22 states in the US. This is more than double the number of cases reported throughout 2024.

**Methods:**

This is a retrospective analysis of measles antibody IgM (IFA/EIA) and IgG (CLIA), and measles PCR (swab and urine) volumes and positivity rates for tests ordered by clinicians between June 2023 through March 2025. De-identified test results were evaluated from a large commercial lab database in the United States. Testing patterns related to age, sex, geography and clinician specialty also were evaluated.

**Results:**

Test volumes for IgM, IgG, and PCR (swab and urine) were 4,772, 1,814,526 and 2221, respectively. Sharp increases in test orders were noted for all measles-related tests beginning in January 2025, although a steady increase in measles IgM was noted beginning in July 2024. PCR testing was 1.39 times more likely to yield a positive result compared to IgM testing (5.0% vs 3.64%, OR=1.39, 95% CI: 1.09 to 1.77), p < 0.05. From January - March 2025, PCR positivity was up 4.5% compared to the same period in 2024. Combined IgM and IgG testing and combined IgM and PCR testing each constituted < 1% of all testing. IgM positivity was highest in the 5-19-year age group (5.1%), whereas PCR positivity was highest between ages 30-39 (6.6%). Overall IgG immunity was 87.4%, with the lowest observed in the < 5-year age group (77%) and the highest in the 66+ year age group (97%). For women of childbearing age (15-49 years), the rate was 86.6%. There were no significant differences in IgG, IgM or PCR positivity rates between sexes. Positivity rates for each geographic region were as follows (IgM vs PCR): South 3.82 vs 6.9, West 2.64 vs 3.09, Northeast 3.79 vs 3.05 and Midwest 2.34 vs 2.38. Testing was ordered mostly by primary care (FP and IM) specialties.

**Conclusion:**

Increases in measles-related testing aligned with recent outbreaks and corresponded to regional positivity rates. Although IgM testing was ordered more than PCR testing, PCR had higher positivity rates. Ordering patterns did not align with current recommendations for the diagnosis of active measles (PCR and IgM) indicating opportunities for provider education. Of note, those >66 years of age demonstrated the greatest level of immunity.

**Disclosures:**

Charles M. Walworth, MD, Labcorp: Employee|Labcorp: Stocks/Bonds (Public Company)|Labcorp: Stocks/Bonds (Public Company) Laura Gillim, PhD, Labcorp: Stocks/Bonds (Public Company) Suzanne Dale, PhD, Labcorp: Stocks/Bonds (Public Company) Sharon K. Martens, MN, Labcorp: Employee David Alfego, PhD, Labcorp: Employee|Labcorp: Employee|Labcorp: Stocks/Bonds (Public Company) Ato Aikins, PhD, Labcorp: Employee

